# Impact of resistance training on body composition and metabolic syndrome variables during androgen deprivation therapy for prostate cancer: a pilot randomized controlled trial

**DOI:** 10.1186/s12885-018-4306-9

**Published:** 2018-04-03

**Authors:** Jacqueline K. Dawson, Tanya B. Dorff, E. Todd Schroeder, Christianne J. Lane, Mitchell E. Gross, Christina M. Dieli-Conwright

**Affiliations:** 1 Divison of Biokinesiology and Physical Therapy, Ostrow School of Dentistry, University of Southern California, 1540 Alcazar Street, CHP-155, Los Angeles, CA 90033 USA; 2 Norris Comprehensive Cancer Center, Keck School of Medicine (KSOM), Los Angeles, CA USA; 3 Department of Preventive Medicine, KSOM, Los Angeles, CA USA; 4 Ellison Institute for Transformative Medicine, KSOM, Los Angeles, CA USA; 5 KSOM, University of Southern California, Los Angeles, CA USA

**Keywords:** Strength training, Prostate cancer, Survivorship, Metabolic syndrome, Sarcopenia, Protein supplementation, Muscle mass, Body fat

## Abstract

**Background:**

Prostate cancer patients on androgen deprivation therapy (ADT) experience adverse effects such as lean mass loss, known as sarcopenia, fat gain, and changes in cardiometabolic factors that increase risk of metabolic syndrome (MetS). Resistance training can increase lean mass, reduce body fat, and improve physical function and quality of life, but no exercise interventions in prostate cancer patients on ADT have concomitantly improved body composition and MetS. This pilot trial investigated 12 weeks of resistance training on body composition and MetS changes in prostate cancer patients on ADT. An exploratory aim examined if a combined approach of training and protein supplementation would elicit greater changes in body composition.

**Methods:**

Prostate cancer patients on ADT were randomized to resistance training and protein supplementation (TRAINPRO), resistance training (TRAIN), protein supplementation (PRO), or control stretching (STRETCH). Exercise groups (EXE = TRAINPRO, TRAIN) performed supervised exercise 3 days per week for 12 weeks, while non-exercise groups (NoEXE = PRO, STRETCH) performed a home-based stretching program. TRAINPRO and PRO received 50 g⋅day^− 1^ of whey protein. The primary outcome was change in lean mass assessed through dual energy x-ray absorptiometry. Secondary outcomes examined changes in sarcopenia, assessed through appendicular skeletal mass (ASM) index (kg/m^2^), body fat %, strength, physical function, quality of life, MetS score and the MetS components of waist circumference, blood pressure, glucose, high-density lipoprotein-cholesterol, and triglyceride levels.

**Results:**

A total of 37 participants were randomized; 32 participated in the intervention (EXE *n* = 13; NoEXE *n* = 19). At baseline, 43.8% of participants were sarcopenic and 40.6% met the criteria for MetS. Post-intervention, EXE significantly improved lean mass (d = 0.9), sarcopenia prevalence (d = 0.8), body fat % (d = 1.1), strength (d = 0.8–3.0), and prostate cancer-specific quality of life (d = 0.9) compared to NoEXE (*p* < 0.05). No significant differences were observed between groups for physical function or MetS-related variables except waist circumference (d = 0.8).

**Conclusions:**

A 12-week resistance training intervention effectively improved sarcopenia, body fat %, strength and quality of life in hypogonadal prostate cancer patients, but did not change MetS or physical function. PRO did not offer additional benefit in improving body composition.

**Trial registration:**

ClinicalTrials.gov: NCT01909440. Registered 24 July 2013.

## Background

Due to marked reductions in testosterone levels, patients receiving androgen deprivation therapy (ADT) for prostate cancer lose approximately 2–4% lean mass in the first year of therapy [1]. This decrease in lean mass, known as sarcopenia, is often accompanied by increases in fat mass [2], decreases in strength [3], physical function [3] and quality of life [3]. Low testosterone levels in prostate cancer patients on ADT have also been associated with the development of the metabolic syndrome (MetS) [4]. While clinical features of ADT-derived MetS differ from the features of classically-defined MetS in type of fat accumulation and high-density lipoprotein-C (HDL-C) response, both are comprised of elevations in blood pressure, triglycerides and glucose levels [5]. Together, the cardiometabolic risk factors that comprise MetS have been associated with insulin resistance [6], and, collectively, these ADT-related changes have been implicated in an increased risk of cardiovascular disease [7].

In healthy adults, exercise has been proposed as a lifestyle modification to enhance body composition, improve MetS components and reduce the risk of cardiovascular disease [8], with results from older adults and insulin resistant patient populations [9] also supporting these recommendations. In prostate cancer patients on ADT, numerous randomized controlled trials (RCTs) utilizing resistance training with or without aerobic training have targeted at least one MetS outcome [10–18], yet no investigations have shown improvements in glucose, triglycerides, or blood pressure levels in exercisers compared to controls. Moreover, although changes in body composition have differed significantly between exercise and control groups, only modest changes in lean mass and body fat have been observed following exercise [10–14]. These findings suggest that the optimal exercise prescription for altering body composition and cardiometabolic variables has yet to be determined in prostate cancer patients on ADT.

Several RCTs have established resistance training as an effective form of exercise for increasing lean mass, strength, physical function and quality of life in men with prostate cancer on ADT [12, 19, 20]. Resistance training may also be an advantageous form of exercise for contributing to fat loss, particularly if substantial metabolic stress is maintained throughout the exercise session through limited rest periods [21]. Previous interventions utilizing resistance training with progressive loads and limited rest periods have demonstrated significant improvements in body composition and cardiometabolic variables in overweight young men [22], middle-aged men [23] and older women [24]. However, prostate cancer patients on ADT may exhibit a blunted response to the anabolic stimulus of exercise due to an older age at the time of diagnosis [25] and treatment-induced testosterone deficiency [26]. The combined use of resistance exercise and protein supplementation (TRAINPRO) has been employed to counter both age- and androgen suppression-related impairments in acute muscle protein synthesis [26], as the synergistic effects of the combined approach are proposed to be greater than either stimulus alone [27]. Taken together, the coupling of hypertrophic resistance training with protein supplementation (PRO) may overcome the blunted response to anabolic stimuli that hypogonadal prostate cancer patients exhibit following ADT administration, thus optimizing lean mass accretion.

Therefore, this pilot study was designed for the primary aim of investigating 12 weeks of supervised resistance training on lean mass in men with prostate cancer on ADT, and to explore the combined approach of resistance training and protein supplementation as an appropriate trial design in this population. For the primary hypothesis, exercise was expected to increase lean mass greater than no exercise. As an exploratory hypothesis, the additive TRAINPRO approach was expected to augment lean mass increases compared to resistance training alone. Secondary aims investigated the effect of exercise on sarcopenia prevalence, body fat, strength, physical function, quality of life, and cardiometabolic markers, including insulin, insulin resistance, and the MetS components of blood pressure, central adiposity, triglycerides, glucose and HDL-C. We hypothesized that exercise would elicit greater improvements in all secondary outcomes compared to no exercise.

## Methods

**Fig. 1:**
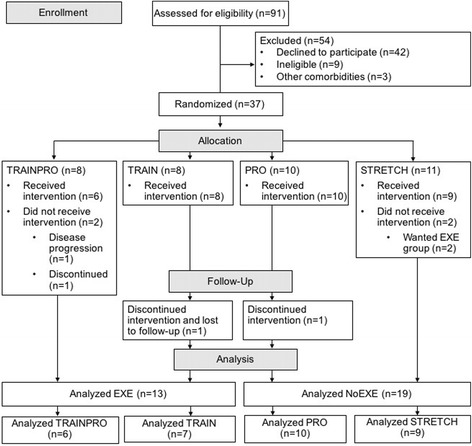
Flow of participants through the trial. EXE, exercise groups; NoEXE, non-exercise groups; TRAINPRO, resistance training protein supplementation group; TRAIN, resistance training; PRO, protein supplementation; STRETCH, control stretching

Details of the study design and methods of this 4-armed randomized controlled pilot study have been previously published [28]. Patients with prostate cancer in Los Angeles, CA were screened for participation between May 2014 and March 2017 (Fig. 1). Participants were randomized 1:1 to one of four study groups: (1) resistance training and protein supplementation (TRAINPRO), (2) resistance training (TRAIN), (3) protein supplementation (PRO), or (4) control stretching (STRETCH). The randomization list was prepared in advance by a biostatistician, and allocation was conducted by the Clinical Investigation Support Office at the University of Southern California (USC) Norris Comprehensive Cancer Center. Trial participants and investigators were not blinded to group allocation.

### Eligibility

Inclusion criteria included current treatment with gonadotrophin-releasing hormone (GnRH) agonist/antagonist with or without anti-androgen for at least 12 weeks, or prior treatment of GnRH agonist/antagonist with serum testosterone concentration < 50 ng·dl^− 1^ at baseline and for the study duration. For patients not currently receiving ADT, an additional screening of testosterone < 50 ng·dl^− 1^ was performed. Exclusion criteria included chemotherapy or radiation within 4 weeks of enrollment, major surgery within 6 months, coronary or vascular event in the last year, or current participation in a structured exercise program. The study was approved by the USC Institutional Review Board (HS-13-00315). All participants provided written consent (ClinicalTrials.gov:NCT01909440).

### Intervention

#### Resistance training program (TRAINPRO and TRAIN groups)

The exercise intervention has been previously described [28]. TRAINPRO and TRAIN groups performed resistance training 3 days per week for 12 weeks with an accredited exercise trainer at the USC Clinical Exercise Research Center. The resistance training program met the American Cancer Society’s (ACS) guidelines for strength training [29] and the Compendium of Physical Activity’s classification for vigorous intensity [30]. Each session was ~ 50 min in duration and began with a 5 min dynamic warmup of body weight exercises that targeted the muscle groups used in the resistance training routine (Table 1). The weekly training volume was divided such that each muscle group was trained twice per week, with lower body and trunk trained on the first day, lower and upper extremities on the second day, and upper body and trunk on the third day. The training routine included 7 machine-based exercises (leg press, leg curl, leg extension, chest press, shoulder press, seated row, lat pulldown) and 3 trunk exercises (plank, hip bridge, dead bug). Because metastatic patients were included, alternative exercises were offered to patients with pre-existing pain due to affected skeletal sites (Table 1). This modular approach has been utilized in previous investigations conducted in patients with bone metastatic disease [31, 32]. Every session concluded with 5 min of static stretching, where hip flexors/extensors were stretched on lower body and total body days, and shoulder flexors/extensors were stretched on upper body and total body days.

**Table 1:** Prescribed and alternative exercises in the resistance training program

Prescribed	Alternative
Dynamic Warmup Exercises	
Free-standing squat	Plie holding bar
Agility ladder forward and lateral shuffle	Agility ladder forward shuffle
Pushup	Elevated pushup on Smith machine bar
Reverse pull-up on Smith machine bar	No alternatives were used
Periodized Resistance Training Exercises	
Leg press	No alternatives were used
Leg curl	Hip bridge on ball + knee extension/flexion
Leg extension	Supine, single leg raise in knee extension
Machine chest press	Resistance band chest press
Smith machine shoulder press	Resistance band lateral raise
Lat pulldown	Resistance band pulldown
Seated row	Resistance band row
Trunk Exercises	
Plank	Elevated plank on Smith machine bar
Single leg hip bridge	Dual leg hip bridge
Dead bug (shoulder/hip flexion/extension)	Deadbug (hip flexion/extension only)

The resistance exercise load was systematically progressed using a periodization model as previously described [28], with 3 sets of each exercise performed and rest periods held constant at 1 min between sets. The periodization model consisted of a 4-week muscular endurance mesocycle and an 8-week hypertrophy mesocycle, where load and repetitions were varied over the course of the intervention. Because brief periods of overreaching were expected due to each load increase, repetitions were tapered by 2 in the second week of each microcycle to maximize adaptations without overtraining [33]. The progression cycle was as follows: Weeks 1–2: 60% 1RM, 15 repetitions; Weeks 3–4: 65–67%, 15–12 repetitions; Weeks 5–6: 70% 1RM, 12–10 repetitions; Weeks 7–8: 75% 1RM, 10–8 repetitions; Weeks 9–10: 80% 1RM, 10–8 repetitions; Weeks 11–12: 83% 1RM, 8 repetitions. Participants were trained to fatigue with each set, where verbal encouragement and light spotting for completion of all repetitions were provided when necessary.

#### Home-based flexibility program

PRO and STRETCH groups performed a home-based flexibility program 3 times per week for 12 weeks, then were offered the exercise program at the end of the study. Each home-based stretching session lasted ~ 5 min and matched the stretches performed by TRAIN and TRAINPRO groups. PRO and STRETCH groups were given a stretching band, a booklet detailing the exercises, and were asked to complete weekly records of flexibility compliance and other exercises performed outside the study with a monetary compensation provided at midpoint and post-intervention.

#### Protein supplementation

TRAINPRO and PRO groups were given 50 g⋅day^− 1^ of whey protein isolate (EnergyFirst®, Manhattan Beach, CA) for 12 weeks. The 50 g daily supplement was divided into two 25 g doses, with each 25 g dose containing 112.5 kcal, 25 g protein (2 g leucine), 0 g fat, and 3.75 g carbohydrate. For TRAINPRO participants, one dose was given immediately after the exercise session to optimize post-exercise anabolic stimuli [34]. Participants completed a protein diary, which was collected on a weekly basis.

### Outcome measures

All endpoints were assessed over 2 visits at baseline and post-intervention. At the first visit, body composition, metabolic syndrome and quality of life outcomes were assessed, while physical fitness was assessed at the second visit. Muscular strength was also assessed at study midpoint.

### Body composition

Dual-energy x-ray absorptiometry (DXA, Lunar GE iDXA, Fairfield, Connecticut) was used to evaluate the primary outcome of lean mass [35], and secondary outcomes of appendicular skeletal mass, fat free mass, fat mass and body fat % following a 12-h fast. Sarcopenia was defined as index (appendicular skeletal mass (kg)/height (m^2^)) < 7.26 kg/m^2^ using the approach of Baumgartner et al. [36], which was chosen due to the use of similar equipment (Lunar GE iDXA) in collecting reference data [37, 38]. Weight was measured to the nearest 0.1 kg (InBody 570, InBody USA, Cerritos, CA). Height was measured on a stadiometer to the nearest 0.5 cm.

### Metabolic syndrome

Participants were considered to present with MetS if criteria for 3 of 5 components were met [6]: 1) waist circumference ≥ 40 in., 2) triglycerides ≥150 mg/dL or medication, 3) HDL-C ≤ 40 mg/dL, 4) systolic blood pressure ≥ 130 mmHg or diastolic blood pressure ≥ 85 or medication, 5) glucose ≥100 mg/dL or medication. Changes in overall MetS status (Yes/No) and a combined score of the 5 MetS variables (MetS sum) were analyzed from baseline to study completion.

Waist circumference was assessed using a tape measure, while blood pressure was measured using an automated device (Welch Allyn, Skaneateles Falls, NY). All serum markers, including testosterone, prostate-specific antigen (PSA), insulin, glucose, HDL-C and triglycerides, were obtained from peripheral blood samples following a 12 h fast and were analyzed by the Diabetes and Obesity Research Institute and Norris Clinical Reference laboratory at USC. Insulin resistance was calculated by the homeostasis model assessment (HOMA-IR = fasting plasma insulin x fasting plasma glucose (mmol/L)/22.5) [39].

### Physical fitness

Physical fitness measures were assessed in the following order: 1) 400 m walk, 2) timed up and go, 3) stair climb and 4) muscular strength.

Functional performance was assessed by the 400 m walk, the timed up and go, and a stair climb [28], with in-house standard error of measurement for each test 1.3%, 0.1% and 0.4%, respectively. Time was recorded to the nearest 0.1 s using a stopwatch, and tests were performed in triplicate except for the 400 m walk.

Prior to the baseline strength testing, two familiarization sessions were performed to acclimate participants to the exercise equipment and increase reliability of the strength measurements [40]. Maximal voluntary strength was estimated through 10RM strength tests at baseline, midpoint and post-intervention for the 6 exercises practiced during the familiarization sessions. Load assignments during the first 6 weeks of the intervention were based on baseline testing values, while loads for the last 6 weeks of the intervention were based on midpoint testing values. To determine the load assignments, 1RM values were calculated from the 10RM load using regression equations for the leg press [41] and chest press [42], while all other exercises used 1RM = *10RM / 0.75* [43]. Participants completed a warm-up load of ~20RM before three attempts were given to reach the final 10RM load. A 2 min rest period was given between attempts. In-house standard error of measurement for leg press and chest press obtained from reliability testing in a subsample of prostate cancer survivors (*n* = 5) are 2.1% and 0.90%, respectively. Participants unable to perform strength testing for a particular exercise due to pre-existing bone lesions or pain were excluded.

### Participant self-reported assessments

Quality of life assessments were completed in person with an investigator available to answer questions if clarification was needed. General and prostate cancer-specific quality of life status were assessed using the Functional Assessment of Cancer Therapy – General (FACT-G) and Prostate (FACT-P), where a higher score indicates better quality of life [44]. Fatigue was assessed using the Brief Fatigue Inventory (BFI), where a lower score indicates less fatigue [45]. Emotional distress was measured using the Center for Epidemiologic Studies – Depression Scale (CES-D), where a lower score indicates the presence of less symptomatology [46].

Physical activity history was assessed at baseline using the International Physical Activity Questionnaire [47]. In addition, PRO and STRETCH groups completed a weekly log of home flexibility program compliance and physical activity performed outside the study. Participants completed a 3-day dietary food intake at baseline and every week during the intervention period, which were analyzed under the supervision of a registered dietician (My Fitness Pal, Under Armour, myfitnesspal.com). Participants who did not meet the minimum recommended daily allowance for adults (0.8 g protein/kg body weight) at baseline were instructed to increase their daily protein intake to this level. Participants were otherwise instructed to maintain customary dietary and physical activity habits throughout the study duration.

### Intervention safety and adherence

The safety of the resistance training program was assessed at every exercise session and testing time point through the identification and grading of adverse events using the common terminology criteria for adverse events (CTCAE v4.3). Protein supplementation tolerability was assessed at every testing time point also using CTCAE (v4.3). In addition, testosterone and the tumor marker prostate-specific antigen (PSA) were measured at baseline and post-intervention and used as circulating safety measures of disease activity. Immunoenzymatic assay was used to measure total testosterone (ELISA, Eagle Biosciences, Nashua, NH) and PSA (MAGPIX, MilliporeSigma, Darmstadt, Germany) by the Diabetes and Obesity Research Institute at USC.

Resistance training attendance was calculated as the number of sessions attended compared to the total number of sessions. Resistance training adherence was calculated as the percentage of exercises performed at the prescribed modality, intensity and volume as specified in the periodization model compared to the total number of exercises. Adherence to protein supplementation and the home-based flexibility program were assessed by the self-reported protein diary and physical activity log, respectively.

### Statistical analyses

Sample size calculations were based on previous studies in prostate cancer patients on ADT, where total sample size was estimated to be 50–60 to detect lean mass changes of 1±1.25 kg - 3±3 kg [12, 20] between exercise and control groups. We estimated that a sample size of 32 participants in a pilot trial would allow us to test the primary outcome hypothesis, inform variance and effect sizes for powering a future RCT, and allow 15% attrition rate.

Patient characteristics were described by 2 groups, EXE (TRAINPRO, TRAIN) and NoEXE (PRO, STRETCH). Distribution of outcomes were evaluated and presented as Mean±SD, while N (%) was used to describe categorical outcomes. Comparisons of baseline patient characteristics between groups were made using t-test or non-parametric corollary for continuous outcomes and χ^2^ test for categorical outcomes. Baseline confounders with a difference of *p* < 0.25 (d = 0.71, large effect size) were considered as additional covariates after testing for collinearity. Adherence and missing data were reported and compared between groups.

For all outcomes, intent-to-treat models compared 12-week change between EXE and NoEXE groups using linear regression adjusted for the a priori covariates of baseline values and PRO, and any additional baseline confounders. Exploratory analyses examined the additive effect of PRO and the potential interaction effect of the two treatments (TRAIN*PRO). To ensure average daily protein intake was consistent with PRO group allocation, sensitivity analyses were performed. While α = 0.05 was used as a cutoff for statistical significance, Cohen’s D effect sizes [48] were calculated to estimate the average difference between and across groups. Analyses were performed in SPSS (V.24, IBM Corp., Armonk, NY).

## Results

Between May 2014 and March 2017, 91 prostate cancer patients were screened for eligibility, 37 agreed to participate, and 32 underwent the intervention (Fig. 1). No significant differences were found between EXE and NoEXE groups for any baseline characteristics (Table 2), although weight, body mass index (BMI), and number of comorbidities demonstrated baseline group differences at *P* < 0.25 and were considered as confounders. After examining multicollinearity, BMI and number of comorbidities were included as covariates in the adjusted analyses. We tested the primary hypothesis that resistance training would improve all outcomes greater than no resistance training, and the exploratory hypothesis that the additive TRAIN*PRO approach would further augment changes in body composition. However, no interaction effect of TRAIN*PRO was found for any outcome. Thus, only data comparing EXE (TRAINPRO, TRAIN) to NoEXE (PRO, STRETCH) are presented.

**Table 2:** Baseline characteristics of exercise and non-exercise groups

	EXE (*n* = 16)	NoEXE (*n* = 19)	*P*
Age (yr)	68.6 ± 8.4	66.3 ± 9.0	0.45
Height (cm)	170.8 ± 8.5	171.6 ± 7.8	0.76
Weight (kg)	85.0 ± 17.8	77.0 ± 12.1	0.13
BMI (kg·m^−2^)	28.5 ± 4.7	26.1 ± 4.0	0.12
ADT duration (mo)	14.6 ± 15.4	12.7 ± 11.6	0.67
Gleason score	7.4 ± 0.9	7.6 ± 0.9	0.50
Time since diagnosis (yr)	4.7 ± 5.1	4.7 ± 4.9	0.99
Testosterone (ng·dl^−1^)	25.0 ± 7.3	6.3 ± 37.4	0.26
PSA (ng·ml^− 1^)	2.1 ± 4.4	0.8 ± 2.4	0.28
Number of comorbidities^a^	2.6 ± 1.9	1.7 ± 1.4	0.13
Number of medications	3.0 ± 2.5	4.1 ± 3.3	0.28
Anti-androgen, n (%)	8 (50%)	12 (63.1%)	0.43
Metastatic disease, n (%)	10 (62.5%)	9 (47.3%)	0.50
Metastases type, n (%)			0.36
Nodal metastases	2 (12.5%)	2 (10.5%)	
Bone metastases	6 (37.5%)	7 (36.8%)	
Other metastases sites^b^	2 (12.5%)	0 (0%)	
Ethnicity, n (%)			0.88
White	8 (50.0%)	11 (57.9%)	
African American	2 (12.5%)	1 (10.5%)	
Asian/Pacific Islander	4 (25.0%)	5 (26.3%)	
Hispanic	2 (12.5%)	2 (10.5%)	
Previous radiation, n (%)	8 (53.3%)	10 (52.6%)	0.97
Previous surgery, n (%)	12 (75.0%)	12 (63.1%)	0.45
Previous chemotherapy, n (%)	2 (12.5%)	2 (10.5%)	0.86
Caloric intake (kcal·day^−1^)	1903.0 ± 629.4	1688.1 ± 376.3	0.26
Protein intake (g·kg^− 1^·day^− 1^)	1.0 ± 0.4	1.1 ± 0.5	0.59
Total physical activity (min·wk.^− 1^)^c^	654.8 ± 789.5	999.1 ± 910.8	0.31
Moderate activity (min·wk.^− 1^)	221.4 ± 210.3	397.3 ± 429.2	0.36
Vigorous activity (min·wk.^− 1^)	144.6 ± 323.8	129.2 ± 151.5	0.86

Data presented as mean ± standard deviation or number of participants (% of participants)

*P*, significance level; EXE, exercise groups (TRAINPRO and TRAIN); NoEXE, non-exercise groups (PRO and STRETCH); ADT, androgen deprivation therapy; PSA, prostate-specific antigen

^a^Cardiovascular disease, hypertension, diabetes, osteoporosis and dyslipidemia

^b^Kidney, liver

^c^Includes light walking, moderate and vigorous activity

### Sarcopenic index and body composition

At baseline, 43.8% of all participants were classified as sarcopenic, while 40.6% of all participants met the criteria for MetS (Table 3). After 12 weeks, the EXE group significantly attenuated sarcopenia prevalence compared to the NoEXE group (*p* = 0.04), but MetS prevalence and sum were consistent pre- and post-intervention in both groups. [Table 3].

**Table 3:** Participants classified as sarcopenic or having the metabolic syndrome at baseline and 12 weeks

	Baseline		Week 12		P*
Variable	n	%	n	%	
Sarcopenia					
EXE	5	38.5	2	15.4	0.04
NoEXE	9	47.4	10	52.6	
MetS					
EXE	6	50.0	6	50.0	0.18
NoEXE	7	36.8	8	42.1	
MetS Sum^a^					
EXE	2.8	1.4	2.8	1.5	0.54
NoEXE	2.1	1.4	2.3	1.6	

Data presented as number of participants, % of participants, except where indicated by ^a^(mean ± standard deviation); *P*, significance level; EXE, exercise groups (TRAINPRO and TRAIN); NoEXE, non-exercise groups (PRO and STRETCH); MetS, metabolic syndrome

*Analysis adjusted for baseline values, protein supplementation, body mass index and number of comorbidities

Body composition outcomes are presented in Table 4. Post-intervention, the EXE group significantly increased muscle mass compared to the NoEXE group as reflected by lean mass (percent change, significance level, effect size; EXE 2.2% vs NoEXE 0.2%, *p* = 0.05, d = 0.9), appendicular skeletal mass (EXE 3.4% vs NoEXE 0.2%, *p* = 0.03, d = 0.9), and sarcopenic index (EXE 3.6% vs NoEXE 0.1%, *p* = 0.02, d = 1.0). Body fat % significantly decreased in EXE (EXE -2.4%, NoEXE 1.4%, *p* = 0.01, d = 1.1). Substituting protein intake for PRO group allocation in the sensitivity analyses did not change the significance for these or any other outcomes. For the exploratory analyses, no significant main effect of PRO or interaction effect of TRAIN*PRO was observed for any of the body composition variables (*P* > 0.05).

**Table 4:** Within- and between-group changes in body composition outcomes over 12 weeks

Outcome	Baseline		Week 12		Within-group mean change over 12 weeks^a^	Between-group mean change over 12 weeks^b^	
	n	Mean ± SD	n	Mean ± SD	Mean (95% CI)	Mean (95% CI)	*P*
Lean Mass (kg)							
EXE	16	48.5 ± 5.4	13	53.2 ± 7.3	1.1 (0.4, 1.8)	1.1 (0.02, 2.2)	0.05
NoEXE	19	51.5 ± 7.6	19	48.6 ± 5.8	0.1 (−0.5, 0.7)		
Appendicular skeletal mass (kg)							
EXE	16	23.5 ± 4.7	13	24.8 ± 4.6	0.8 (0.4, 1.2)	0.7 (0.1, 1.3)	0.03
NoEXE	19	21.5 ± 2.6	19	21.6 ± 2.9	0.1 (−0.3, 0.4)		
Sarcopenic index (kg/m^2^)							
EXE	16	8.1 ± 1.5	13	8.5 ± 1.6	0.3 (0.2, 0.4)	0.3 (0.1, 0.5)	0.02
NoEXE	19	7.3 ± 0.8	19	7.3 ± 0.9	0.01 (−0.1, 0.1)		
Fat Free Mass (kg)							
EXE	16	54.6 ± 7.9	13	56.4 ± 7.5	1.0 (0.4, 1.8)	1.1 (0.1, 2.2)	0.04
NoEXE	19	51.4 ± 5.7	19	51.5 ± 6.0	0.1 (−0.5, 0.7)		
Body Fat (%)							
EXE	16	36.8 ± 5.1	13	35.9 ± 5.6	−0.9 (−1.8, − 0.01)	− 1.7 (−2.9, − 0.6)	0.01
NoEXE	19	33.9 ± 5.9	19	34.5 ± 5.3	0.5 (−0.2, 1.2)		
Fat Mass (kg)							
EXE	16	30.3 ± 10.5	13	31.2 ± 11.6	−0.3 (−1.2, 0.6)	−0.7 (−2.0, 0.6)	0.26
NoEXE	19	25.6 ± 8.3	19	26.2 ± 8.0	0.6 (−1.5, 1.3)		
Total Mass (kg)							
EXE	16	85.0 ± 17.8	13	87.5 ± 18.8	0.8 (−0.5, 2.0)	0.3 (−1.5, 2.1)	0.75
NoEXE	19	77.0 12.1	19	77.7 ± 12.4	0.7 (−0.3, 1.7)		

Data presented as mean ± standard deviation. *P*, significance level; EXE, exercise groups (TRAINPRO and TRAIN); NoEXE, non-exercise groups (PRO and STRETCH)

^a^EXE relative to NoEXE adjusted for baseline values

^b^EXE relative to NoEXE adjusted for baseline values, protein supplementation, body mass index and number of comorbidities

### Metabolic syndrome

MetS-related outcomes are presented in Table 5. Three EXE participants did not perform baseline MetS testing as 2 withdrew after randomization (TRAINPRO), and 1 (TRAIN) refused the blood draw, then withdrew shortly after. After 12 weeks, no significant differences were observed between EXE and NoEXE for insulin, HOMA-IR or any MetS variables except for waist circumference, which decreased significantly in EXE compared to NoEXE (EXE -1.1%, NoEXE 2.0%, *p* = 0.013, d = 0.9).

**Table 5:** Within- and between-group changes in metabolic syndrome variables over 12 weeks

	Baseline		Week 12		Within-group mean change over 12 weeks^a^	Between-group mean change over 12 weeks^b^	
Outcome	n	Mean ± SD	n	Mean ± SD	Mean (95% CI)	Mean (95% CI)	*P*
Waist circumference (cm)							
EXE	13	106.8 ± 14.2	13	105.6 ± 14.4	−1.2 (−3.3, − 1.0)	−3.7 (−6.6, − 0.8)	0.01
NoEXE	19	97.9 ± 10.1	19	99.6 ± 12.4	2.0 (0.2, 3.7)		
Systolic blood pressure (mm Hg)							
EXE	13	122.2 ± 14.2	13	122.4 ± 15.3	−1.4 (− 8.5, 5.7)	−5.1 (− 15.5, 5.3)	0.32
NoEXE	19	128.8 ± 13.0	19	129.8 ± 14.0	2.1 (−3.8, 7.9)		
Diastolic blood pressure (mm Hg)							
EXE	13	76.9 ± 9.2	13	75.4 ± 8.9	−1.9 (−6.3, 2.5)	− 0.5 (− 6.8, 5.8)	0.87
NoEXE	19	79 ± 5.1	19	79.0 ± 8.6	0.3 (−3.3, 4.0)		
HDL-C (mg/dL)							
EXE	13	46.5 ± 12.2	12	49.0 ± 19.0	3.8 (−1.9, 9.6)	6.1 (− 1.6, 13.8)	0.11
NoEXE	19	61.3 ± 16.3	19	61.5 ± 18.4	− 0.3 (−4.8, 4.1)		
Triglycerides (mg/dL)							
EXE	13	152.9 ± 65.4	12	148.7 ± 42.4	−3.7 (−31.9, 24.4)	−6.7 (− 46.7, 33.2)	0.73
NoEXE	19	131.63 ± 63.7	19	130.2 ± 79.5	−3.6 (−25.8, 18.6)		
Glucose (mg/dL)							
EXE	13	106.1 ± 33.0	13	103.7 ± 18.1	−4.0 (−9.8, 1.8)	− 4.7 (− 13.2, 3.8)	0.27
NoEXE	19	104.5 ± 20.3	19	104.7 ± 20.4	−0.4 (−5.2, 4.4)		
Insulin (mU/L)							
EXE	13	7.9 ± 7.6	12	5.5 ± 2.7	−1.3 (−5.6, 2.9)	−2.0 (−8.1, 4.0)	0.50
NoEXE	19	4.9 ± 4.7	19	6.6 ± 9.2	0.9 (−2.4, 4.3)		
HOMA-IR							
EXE	13	2.6 ± 4.2	12	1.4 ± 0.7	−0.6 (− 1.9, 0.6)	−0.7 (−2.5, 1.0)	0.39
NoEXE	19	1.3 ± 1.5	19	1.8 ± 2.6	−0.01(−1.0, 1.0)		

Data presented as mean ± standard deviation. *P*, significance level; EXE, exercise groups (TRAINPRO and TRAIN); NoEXE, non-exercise groups (PRO and STRETCH); HDL-C; high-density lipoprotein cholesterol; HOMA-IR, homeostatic model of assessment of insulin resistance

^a^EXE relative to NoEXE adjusted for baseline values

^b^EXE relative to NoEXE adjusted for baseline values, protein supplementation, body mass index and number of comorbidities

### Muscular strength and physical function

Pre-existing knee pain prevented participants from performing the leg press (EXE *n* = 1), leg extension (NoEXE *n* = 3), 400 m walk (EXE *n* = 2, NoEXE *n* = 4), stair climb (EXE *n* = 2, NoEXE *n* = 4) and timed up and go tests (EXE *n* = 1, NoEXE *n* = 3). Pre-existing shoulder pain prevented participants from performing the chest press (NoEXE *n* = 1) and shoulder press tests (NoEXE *n* = 1). Muscular strength and physical function results are shown in Table 6. The EXE group significantly increased strength compared to NoEXE in leg press (EXE 61.9% vs NoEXE − 6.7%, *p* < 0.01, d = 1.9), chest press (EXE 59.3% vs NoEXE 6.8%, *p* = 0.05, d = 0.8), seated row (EXE 36.5%, 3.2%, *p* < 0.01, d = 3.0), leg curl (EXE 30.1%, NoEXE 0.2% *p* < 0.01, d = 1.2), shoulder press (EXE 80.7%, NoEXE 5.8%, *p* = 0.01, d = 1.5), and leg extension (EXE 50.7%, NoEXE 0.3%, *p* < 0.01, d = 1.4). Although not statistically significant, after 12 weeks, EXE exhibited improvements compared to the NoEXE in time to completion for the 400 m walk (EXE − 8.4% vs NoEXE 0.7%, *p* = 0.13, d = 0.7), timed up and go (EXE − 15.0% vs NoEXE − 4.9%, *p* = 0.07, d = 0.8) and stair climb (EXE − 13.5% vs NoEXE 0.9%, *p* = 0.14, d = 0.7). No main effect of PRO or interaction effect of TRAIN*PRO were observed for any of the strength or physical function outcomes (*p* > 0.05).

**Table 6:** Within- and between-group changes in muscular strength and physical function over 12 weeks

	Baseline		Week 12		Within-group mean change over 12 weeks^b^	Between-group mean change over 12 weeks^c^	
Outcome	n	Mean ± SD	n	Mean ± SD	Mean (95% CI)	Mean (95% CI)	*P*
Leg press (kg)^a^							
EXE	12	175.9 ± 78.2	12	288.7 ± 80.1	108.8 (74.1, 143.6)	106.5 (59.8, 153.1)	< 0.01
NoEXE	19	137.7 ± 68.6	19	130.3 ± 82.4	−9.2 (−35.0, 16.7)		
Chest press (kg) ^a^							
EXE	13	25.5 ± 10.9	13	40.9 ± 16.0	15.1 (7.3, 23.0)	11.1 (0.1, 22.0)	0.048
NoEXE	18	26.1 ± 13.3	18	26.4 ± 16.3	1.8 (−4.6, 8.2)		
Seated row (kg)^a^							
EXE	13	59.3 ± 12.9	13	81.9 ± 18.3	21.6 (17.4, 25.9)	20.1 (14.6, 25.7)	< 0.01
NoEXE	19	52.4 ± 13.3	19	50.4 ± 20.6	1.7 (−2.0, 5.4)		
Leg curl (kg) ^a^							
EXE	13	81.4 ± 22.1	12	108.6 ± 25.4	24.5 (19.5, 29.6)	25.9 (7.7, 44.0)	< 0.01
NoEXE	18	73.6 ± 17.5	18	68.1 ± 24.5	0.2 (−4.4, 4.1)		
Shoulder press (kg) ^a^							
EXE	13	13.5 ± 9.0	12	27.3 ± 13.1	10.9 (6.7, 15.0)	53.7 (18.6, 88.8)	< 0.01
NoEXE	18	12.1 ± 9.6	18	12.6 ± 11.1	0.7 (−2.5, 4.0)		
Leg extension (kg) ^a^							
EXE	13	110.8 ± 43.6	13	178.4 ± 42.7	56.2 (36.0, 76.4)	8.4 (2.7, 14.2)	< 0.01
NoEXE	16	90.7 ± 24.4	16	89.4 ± 41.7	0.3 (−17.4, 16.9)		
400 m walk (s)							
EXE	11	248.5 ± 50.0	11	227.7 ± 46.7	−20.9 (−3.5, 45.4)	−32.3 (−74.4, 9.8)	0.13
NoEXE	15	257.9 ± 60.2	15	257.6 ± 79.0	1.9 (−23.2, 19.4)		
Timed up and go (s)							
EXE	12	5.5 ± 2.3	12	4.7 ± 1.8	−0.8 (−1.4, −0.3)	−0.8 (− 1.6, 0.1)	0.07
NoEXE	16	5.3 ± 1.8	16	5.6 ± 2.6	−0.3 (− 0.7, 0.2)		
Stair climb (s)							
EXE	11	2.2 ± 0.7	11	1.9 ± 0.5	−0.3 (−0.6, 0.03)	−0.4(−1.0, 0.1)	0.14
NoEXE	15	2.2 ± 0.8	15	2.3 ± 1.2	0.02 (−0.3, 0.3)		

Data presented as mean ± standard deviation. *P*, significance level; EXE, exercise groups (TRAINPRO and TRAIN); NoEXE, non-exercise groups (PRO and STRETCH)

^a^Strength values presented as estimated 1-repetition maximum (RM) values calculated from 10-RM tests

^b^EXE relative to NoEXE adjusted for baseline values

^c^EXE relative to NoEXE adjusted for baseline values, protein supplementation, body mass index and number of comorbidities

### Quality of life

Six participants (EXE n = 1, NoEXE *n* = 5) declined to answer the quality of life questionnaires. Significantly greater improvements were observed in EXE compared to NoEXE for general (FACT-G, EXE 12.5% vs NoEXE − 3.0%, *p* = 0.048, d = 0.9) and prostate cancer-specific quality of life (FACT-P, EXE 12.3% vs NoEXE − 2.8%, p = 0.04, d = 0.9) (Table 7). Although EXE exhibited greater improvements in fatigue as measured by the BFI (EXE -26.4% vs NoEXE − 6.2%, *p* = 0.31, d = 0.4) and depression as measured by the CES-D (EXE -16.3% vs NoEXE − 11.9%, *p* = 0.75, d = 0.4), no significant differences were observed between groups. No significant main effect of PRO or interaction effect of TRAIN*PRO was observed for any of the quality of life measures (*P* > 0.05).

**Table 7:** Within- and between-group changes in quality of life over 12 weeks

	Baseline		Week 12		Within-group mean change over 12 weeks^a^	Between-group mean change over 12 weeks^b^	
Outcome	n	Mean ± SD	n	Mean ± SD	Mean (95% CI)	Mean (95% CI)	*P*
Prostate cancer symptoms (FACT-P)							
EXE	12	95.1 ± 19.1	12	106.3 ± 24.9	11.7 (0.7, 22.8)	18.0 (1.2, 34.9)	0.04
NoEXE	14	106.1 ± 30.4	14	107.7 ± 30.8	−3.0 (−12.3, 6.2)		
General (FACT-G)							
EXE	12	69.4 ± 14.1	12	78.4 ± 15.4	8.7 (1.3, 16.2)	11.2 (0.1, 22.2)	0.048
NoEXE	14	83.2 ± 15.5	14	80.6 ± 17.5	−2.5 (−9.2, 4.3)		
Fatigue (BFI)							
EXE	12	4.2 ± 2.4	12	2.5 ± 2.0	−1.1 (−2.3, 0.1)	− 0.8 (− 2.6, 1.0)	0.36
NoEXE	14	3.2 ± 2.9	14	2.6 ± 2.8	−0.2 (−1.4, 0.9)		
Depression (CES-D)							
EXE	12	15.3 ± 9.8	12	11.5 ± 6.5	−2.5 (−6.7, 1.8)	−2.8 (−8.9, 3.3)	0.34
NoEXE	14	12.6 ± 11.2	14	9.3 ± 8.6	−1.5 (−5.5, 2.5)		

Data presented as mean ± standard deviation. *P*, significance level; EXE, exercise groups (TRAINPRO and TRAIN); NoEXE, non-exercise groups (PRO and STRETCH)

^a^EXE relative to NoEXE adjusted for baseline values

^b^EXE relative to NoEXE adjusted for baseline values, protein supplementation, body mass index and number of comorbidities

### Dietary intake

Dietary intake was not significantly different between EXE and NoEXE groups over 12 weeks (*P* > 0.05, Table 8). PRO groups significantly increased protein intake from baseline to intervention (*p* = 0.01), and protein intake in PRO was significantly greater compared to no PRO over the 12-week intervention (*P* = 0.048). A significant interaction effect of TRAIN*PRO was observed for protein intake (*p* = 0.04), but not for protein intake normalized to body weight, carbohydrate, fat, or caloric intake.

**Table 8:** Average daily dietary values during the 12-week intervention for all groups

	Baseline		Weeks 1–12						
					TRAIN*PRO	EXE vs. NoEXE		PRO vs. NoPRO	
Outcome	n	Mean ± SD	n	Mean ± SD	*P* ^*†*^	Group difference in mean change (95% CI)	*P* ^*^	Group difference in mean change (95% CI)	*P* ^**^
Protein (g⋅day^− 1^)					0.04	4.6 (−9.0, 18.1)	0.49	13.0 (0.1, 25.9)	0.048
TRAINPRO	8	93.9 ± 34.3	6	118.1 ± 30.2					
TRAIN	8	74.2 ± 15.6	7	83.1 ± 13.9					
PRO	10	85.5 ± 44.4	10	99.5 ± 38.6					
STRETCH	9	76.5 ± 30.6	9	87.8 ± 33.7					
Protein (g ·kg^−1^·day^−1^)					0.14	0.0 (−0.2, 0.2)	0.77	0.4 (0.1, 0.7)	0.01
TRAINPRO	8	1.1 ± 0.5	6	1.4 ± 0.5					
TRAIN	8	1.1 ± 0.7	7	1.2 ± 0.6					
PRO	10	0.9 ± 0.2	10	1.0 ± 0.2					
STRETCH	9	1.0 ± 0.3	9	1.1 ± 0.6					
Carbohydrates (g⋅day^−1^)					0.12	37.4 (−1.8, 76.6)	0.06	−5.6 (−44.4, 33.2)	0.77
TRAINPRO	8	239.3 ± 78.6	6	217.8 ± 74.1					
TRAIN	8	222.0 ± 94.8	7	243.1 ± 30.4					
PRO	10	190.2 ± 65.2	10	184.6 ± 73.5					
STRETCH	9	184.6 ± 57.4	9	162.0 ± 38.8					
Total fat (g⋅day^−1^)					0.82	5.1 (−7.9, 18.1)	0.43	−5.9 (−18.6, 6.8)	0.35
TRAINPRO	8	70.8 ± 55.4	6	77.8 ± 53.4					
TRAIN	8	78.7 ± 27.6	7	77.4 ± 16.3					
PRO	10	71.1 ± 19.1	10	63.1 ± 23.5					
STRETCH	9	53.5 ± 12.9	9	61.1 ± 17.2					
Total energy (kcal⋅day^−1^)					0.73	107.0 (−85.4, 299.4)	0.27	−24.2 (− 251.1, 202.6)	0.83
TRAINPRO	8	1976 ± 708	6	2062 ± 753					
TRAIN	8	1866 ± 636	7	1988 ± 193					
PRO	10	1784 ± 447	10	1778 ± 479					
STRETCH	9	1561 ± 284	9	1624 ± 330					

Data presented as mean ± standard deviation. P, significance level; EXE, exercise groups (TRAINPRO and TRAIN); NoEXE, non-exercise groups (PRO and STRETCH); PRO, protein supplementation groups (TRAINPRO and PRO); NoPRO, non-protein supplementation groups (TRAIN and STRETCH)

^*†*^Interaction effect of (TRAIN*PRO)

^*^Main effect of EXE adjusted for baseline values

^**^Main effect of PRO adjusted for baseline values

### Intervention safety and adherence

Following baseline testing, three participants withdrew from the EXE groups before beginning the exercise intervention (Fig. 1) due to disease progression (*n* = 1, TRAINPRO) or a change of mind (n = 1, TRAINPRO; n = 1, TRAIN). No adverse events occurred during the exercise sessions or due to the protein supplementation. Attendance at the 36 exercise sessions by the EXE groups was 93.8 ± 2.0%, with 77% of TRAIN and TRAINPRO participants completing all 36 sessions. Sessions were missed for personal reasons/schedule conflicts in 3 participants. Adherence to the exercise program using the exercises, intensities and volumes as prescribed in the periodization model was 88.3 ± 16.0% (Table 9). Exercise modifications occurred due to pre-existing knee pain (*n* = 1), musculoskeletal injuries sustained at home during the intervention period (*n* = 2), or avoidance of bone metastatic regions (*n* = 2). Deviations from the prescribed volume or intensity most commonly occurred due to patient-reported fatigue. One participant in the PRO group refused to take the protein after 2 weeks, but remained in the study. Protein supplementation adherence in the TRAINPRO and PRO groups was 72.0 ± 22.8%, with 120.9 ± 58.1 doses of 168 total ingested. Adherence to the home-based flexibility program in PRO and STRETCH was 79.0 ± 4.1%, with 28.4 ± 10.4 of 36 sessions completed. No significant changes were observed post-intervention in the circulating safety markers PSA or testosterone for EXE compared to NoEXE (PSA, *p* = 0.52; testosterone, *p* = 0.79,) or PRO compared to no protein supplementation (NoPRO) (PSA, *p* = 0.25; testosterone, *p* = 0.09).

**Table 9:** Resistance exercise intervention adherence

Measure	EXE (*n* = 13)	EXE with bone metastases (*n* = 6)
Exercise session attendance (out of 36 sessions)	33.8 ± 5.2	35.0 ± 2.4
All exercises adherence (%)^a^	88.3 ± 16.0	91.4 ± 25.7
Lower body exercise adherence (%)^a^	85.6 ± 7.7	95.8 ± 4.0
Upper body exercise adherence (%)^a^	88.2 ± 7.7	84.9 ± 20.5
Trunk exercise adherence (%)^a^	93.3 ± 2.8	94.2 ± 4.9

EXE, exercise groups (TRAINPRO and TRAIN)

^a^Adherence calculated as % of total exercises completed using the type, intensity and volume as prescribed in the periodization model

## Discussion

This randomized controlled pilot study demonstrated that 12 weeks of vigorous-intensity resistance training could counter treatment-related sarcopenia and fat gain, increase upper and lower extremity strength, and enhance prostate cancer-specific quality of life in prostate cancer patients on ADT. However, no significant improvements in MetS or physical function were found. Twice per day protein supplementation (50 g⋅day^− 1^) did not further enhance the effects of resistance training on body composition, strength or quality of life.

This study is the first to classify sarcopenia in men with prostate cancer on ADT using a standardized index [36], and to establish improvement via exercise. Nearly 44% of study participants were classified as sarcopenic at baseline, and though prevalence increased in NoEXE, sarcopenia was significantly reduced in EXE. This finding is particularly relevant considering that the prevalence of sarcopenia in prostate cancer patients on ADT is much greater than in age- and gender-matched individuals in the general population (13.5%) [38]. Significant increases in fat free mass, lean mass and appendicular skeletal mass were also observed in EXE compared to NoEXE post-intervention. As no differences in daily protein intake or caloric consumption were found between EXE and NoEXE groups, the lean mass increase observed in EXE may be attributed to the hypertrophic nature of the resistance training rather than to dietary differences between groups. The high adherence of the EXE group (93.8%) may have also contributed to the training program efficacy, as a previous intervention in prostate cancer patients on ADT reported 79% adherence without significant changes in lean mass [14].

The high adherence in the present study may reflect our use of the modular exercise program concept [32], where an alternative set of exercises that minimize compressive loads to bone metastatic lesions while targeting the same muscle groups in the originally prescribed program is employed. Similar to findings in a previous investigation involving prostate cancer patients with bone metastases [31], our program was well-tolerated and well-attended, with exercise session attendance ~ 94%, and overall EXE group adherence to the specific movements, loads and volumes in the originally prescribed program > 85%. This suggests that machine-based exercises at moderate-to-vigorous loads and limited rest periods are safe and tolerable for patients with metastatic disease, especially if the exercises begin at a lower intensity and are gradually progressed.

Substantial improvements in upper and lower extremity estimated strength were observed post-intervention in the EXE groups compared to NoEXE, with large effect sizes supporting the differences between groups. These findings are consistent with previous RCTs utilizing progressive resistance training in men with prostate cancer on ADT that demonstrated increases in strength following 12 weeks – 1 year [12, 19, 20, 49]. However, we did not observe significant differences between EXE and NoEXE groups in functional performance measures, including the 400 m walk, timed up and go, and stair climb. Yet, effect sizes for the differences were moderate-to-large, suggesting that the number of participants (3–6) unable to perform the functional performance measures may have influenced the statistical significance of the results. Significant improvements have been reported in the stair climb following 16 weeks of seated machine-based resistance exercise [20] and in the 400 m walk following a combined program of 12 weeks of machine-based resistance and aerobic exercise [10]. Thus, despite the use of functional movements (i.e. squats, agility ladder) in the dynamic warmup of our program, a duration longer than 12 weeks and inclusion of aerobic exercise may be necessary to improve functional performance in men with prostate cancer on ADT.

Similarly, the resistance training intervention did not change MetS score or yield significant improvements in any cardiometabolic variables except for waist circumference. Importantly, this study was not powered to detect changes in MetS, although the lack of improvement is surprising considering that resistance training has been shown to enhance insulin sensitivity in type II diabetics [50], while resistance plus aerobic training has been shown to reduce MetS criteria in dyslipidemic [51] and type II diabetic adults [52]. One explanation for the discrepancy between the present study and non-cancer study findings may relate to the overall greater exercise intensity (vigorous-to-high) and volume (120–180 min/week) performed in the non-cancer studies [51, 52]. In addition, the lack of aerobic training and modest prevalence of MetS in the study sample at baseline might have impacted our ability to further detect cardiometabolic changes.

Quality of life, as measured by the FACT-P, significantly improved for the EXE group, increasing > 14 points over the NoEXE group. As a difference in total FACT-P score of 6–10 points is considered clinically meaningful [53], the 14-point difference and large effect size between EXE and NoEXE groups suggests that the exercise intervention was beneficial in improving patient well-being. On the other hand, no effect of exercise was observed for fatigue or depression as measured by the BFI or CES-D, respectively. Other resistance exercise trials have reported improvements in fatigue as measured by the Functional Assessment of Cancer Therapy – Fatigue (FACT-F) scale [14, 54]. Thus, it is plausible that the FACT-F may be more sensitive to exercise-induced improvements, although the vigorous nature of the periodized resistance training program may have negatively influenced fatigue levels, which were reflected in the BFI scores.

This study was the first to investigate the combined effect of resistance exercise and short-term protein supplementation in prostate cancer survivors on ADT, yet no benefit of protein supplementation was observed. However, protein supplementation was not associated with significant adverse events or changes in testosterone or PSA, suggesting that it is safe and tolerated in prostate cancer survivors on ADT. The lack of additional improvements due to protein supplementation may be attributed to differences in dosage, as participants in the present study consumed less (2 × 25 g⋅day^− 1^) then a previous investigation where protein consumption (40 g⋅day^− 1^ in a single sitting) induced significant elevations in muscle protein synthesis in prostate cancer participants on ADT [26]. When normalized to body weight, 1 × 40 g⋅day^− 1^ reflects ~ 0.4 g protein kg^− 1^ body weight [26], which was greater than the ~ 0.3 g protein kg^− 1^ body weight used in the present study. A second contributing factor may be the failure of the TRAINPRO and PRO groups to supplement 50⋅day^− 1^ above their habitual diets as prescribed. Dietary records revealed a net protein increase of only ~ 19 g⋅day^− 1^ in PRO groups instead of the expected 50⋅day^− 1^ increase. Although this increase represented a significantly greater protein intake in the PRO groups compared to baseline and non PRO groups, it is well below the amount proposed to work synergistically with resistance exercise to maximally stimulate skeletal muscle adaptations. These observations suggest that a standardized diet is necessary to ensure that protein supplementation is occurring over habitual dietary protein intake, and that higher amounts of protein at each dosing are likely warranted to achieve a synergistic effect of TRAINPRO.

There are several novel aspects to this study that serve as its strengths. This study classified prostate cancer patients using a standardized sarcopenic index, allowing for direct comparison with non-cancer population studies that also use this index. In addition, this study evaluated all metabolic syndrome risk factors, including metabolic syndrome score, in prostate cancer patients on ADT following an exercise program, thus contributing to the body of evidence surrounding the mitigation of ADT side effects. The use of periodization in the resistance training program provided a defined method of progression, allowing for future replicability of these results. Finally, this study was the first to use short-term protein supplementation to augment the effects of resistance training in hypogonadal cancer patients. This study also had important limitations. While the sample size was adequate to detect changes in the primary outcome of lean mass between EXE and NoEXE groups, this study was underpowered to detect changes in any outcome using a 4-armed design. In addition, our approach to classifying sarcopenia was based solely on muscle mass, rather than the three-outcome criteria of muscle mass, handgrip strength and gait speed developed by the European Working Group on Sarcopenia in Older People (EWGSOP) [55]. Because no caloric control or standardized diet was provided to participants, the PRO groups failed to consistently supplement with 50 g⋅day^− 1^ of protein over their habitual dietary patterns. While a higher dose of protein might have been necessary to elicit greater improvements in all outcomes, as this was the first investigation to employ a short-term protein supplementation intervention in prostate cancer patients, a conservative supplementation approach was used. Future investigations wishing to employ higher doses of protein intake may need to consider subtle effects of long-term supplementation on kidney function or other complicating factors. Other limitations include a non-sedentary control group with high baseline physical activity levels that might have blunted the effects of resistance training, the use of physical activity self-report rather than accelerometery, the use of supervised exercise that limits the generalization of these results to home-based or group-based settings, and an ethnically-homogenous pool of participants that limits the generalization of these results to minority or underrepresented groups.

## Conclusions

This study provides preliminary evidence supporting a vigorous intensity resistance exercise program in improving sarcopenia, body fat, muscular strength and quality of life in hypogonadal prostate cancer patients. Given the prevalence of sarcopenia and MetS observed in this sample, exercise interventions that target both skeletal muscle loss and cardiometabolic risk factors are needed to address the chronic metabolic complications of ADT.

## Abbreviations

ACS: American Cancer Society
ADT: Androgen deprivation therapy
ASM: Appendicular skeletal mass
EXE: Exercise groups
GnRH: Gonadotrophin-releasing hormone
HDL-C: High-density lipoprotein cholesterol
HOMA-IR: Homeostasis model assessment of insulin resistance
MetS: Metabolic syndrome
NoEXE: Non-exercise groups
PRO: Protein supplementation group
PSA: Prostate-specific antigen
RCT: Randomized controlled trial
STRETCH: Stretching group
TRAIN: Resistance training group
TRAINPRO: Resistance training and protein supplementation group
